# One Idea

**Published:** 1856-12

**Authors:** 


### ONE IDEA.

The man of single purpose, or one idea, as it is commonly denominated, is the object of sport and ridicule, by mankind generally. This may arise from the fact that oftentimes men who concentrate the mind upon one object for a long time, lose their ability and disposition to efficiently exert themselves in any other channel; or they endeavor to make every thing bend and become subservient to their favorite object.

Now, though we do not like to see any one blindly riding a hobby, not knowing whither it is carrying him, we do like to see the man of single purpose. One-idea-ism has become almost a term of reproach; and yet almost every body recognizes the fact that when an intelligent man fixes his mind upon one object, and pursues it perseveringly for a long time, something of interest or value *will be accomplished.* Such a course, without general intelligence, would avail but little.

What would have been accomplished by Watt, Newton, or Morse, if their minds had been alike employed in the various fields of science which lay in their view? Certainly, so far as they are concerned, we would not now have the steam engine, steam navigation, or the electric telegraph. These are the products of *men of one idea.* It is true that these have all been much improved since their introduction; but it has been by men of similar mental dispositions.

Now, we do not maintain that a man should know but one thing. On the contrary, we contend for the largest amount of intelligence upon all scientific and useful subjects; and it is only in the possession of these that the mind can act most efficiently. A thorough knowledge of kindred sciences will enable any one to draw upon them, and make them tributary to his favorite purpose. In the architect we may find an illustration. His single purpose is to build a house; but then, in order to do this properly, he must know what materials he requires, the nature of these materials, and be able to judge of their quality, of the amount he may require in any given case, and the sources from whence he may obtain them; and then, the best manner of adjusting them. The brick should not be put in place of the wood, nor the glass in the place of the stone. Now, it is not expected that the man should go to work, in order to ascertain the fitness of these different materials for any given position, and place them in all different possible positions. His knowledge of their nature, independent of house-building, is sufficient to prevent his making any mistakes in regard to their adaptation. But, suppose he did not possess this knowledge of collateral subjects, he would, doubtless, spend a great amount of time and labor in experimenting, to ascertain the adaptation and fitness of the materials he is about to use.

When once in possession of all the collateral branches of science, the only method of making that knowledge efficient is to concentrate the mind upon one object, and draw upon all these departments for aid; and they will be prodigal of that aid, just in proportion to their familiarity with them. Hence, we contend for the largest possible amount of both literary and scientific knowledge for the dentist, that these may be made subservient to his art and science. Now, notwithstanding the remarks just made, we hope to see the *one idea* principle carried out in dental practice. After being posted up perfectly, then let each one, as his inclination may direct, select some special point of practice, and let it be his province to develop, improve, and bring it to perfection, and then make the result known. It is not necessary that every thing else should be forgotten, or any thing even neglected; but keep the one point the prominent one in the mind. We are encouraged to make these remarks from what we have seen. A few, though *very few*, have been pursuing this course, and the result is manifest—indeed, it is one great means of the advancement of the profession, and one which, when fully adopted, will produce a corresponding progression in dental practice.

We observe all along through the routine of dental practice, points, numerous and prominent, that require development, improvement, change, and, occasionally, obliteration. We would thank somebody to *wipe* out some things that are occasionally met with in dental practice.

Let each take a small field, and bring *it* up to the *highest* point of improvement; and, when that is accomplished, take another, and, after a while, the whole tract will be under most glorious cultivation. T.

